# Comparison of clinical efficacy of two approaches for endoscopic lumbar fusion surgery in the treatment of single-segment lumbar spondylolisthesis

**DOI:** 10.3389/fsurg.2025.1588997

**Published:** 2025-09-23

**Authors:** Kun Li, Hang Lin, Zhibin Zhang, Xiangyu Meng

**Affiliations:** ^1^ Graduate School of Xinjiang Medical University, Urumqi, China; ^2^ Minimally Invasive Spine Surgery, Sixth Affiliated Hospital of Xinjiang Medical University, Urumqi, China

**Keywords:** low back pain, lumbar spondylolisthesis, spinal degenerative diseases, unilateral biportal endoscopy, percutaneous endoscopic, spinal fusion

## Abstract

**Objectives:**

Lumbar Spondylolisthesis (LSP) is a frequently encountered degenerative disorder of the spine. Unilateral biportal endoscopy (UBE) and percutaneous endoscopy (PE) have each shown promising initial results in managing this condition. This study aimed to compare the clinical efficacy of unilateral biportal endoscopic lumbar interbody fusion (UBE-LIF) and percutaneous endoscopic lumbar interbody fusion (PE-LIF) in treating single-level LSP, with the objective of providing high-quality evidence to support clinical decision-making.

**Methods:**

A retrospective analysis was conducted on clinical records from 118 patients diagnosed with single-segment LSP who were treated at the Sixth Affiliated Hospital of Xinjiang Medical University between June 2021 and August 2023. Participants were categorized into two groups: UBE-LIF (*n* = 61) and PE-LIF (*n* = 57). Parameters assessed included operative duration, intraoperative blood loss, and postoperative levels of serum biomarkers, creatine kinase (CK) and C-reactive protein (CRP), measured on the third day following surgery. Furthermore, evaluations were made using the visual analog scale (VAS) for back and leg pain, and the Oswestry Disability Index (ODI), at baseline, as well as at 3 days, 3 months, 6 months, and 1 year postoperatively. Radiographic fusion rates and incidences of postoperative complications were also compared.

**Results:**

All procedures were successfully completed. Intraoperative blood loss was slightly higher in the PE-LIF group, without significant difference (*P* = 0.568). The UBE-LIF group had a shorter operative duration (*P* < 0.001). On postoperative day 3, the UBE-LIF group exhibited significantly lower CRP levels compared to the PE-LIF group (*P* = 0.009). Both treatment groups demonstrated marked improvement in VAS and ODI scores across all follow-up periods, with no statistically significant intergroup differences at any time point (*P* > 0.05). Fusion rates and the incidence of postoperative complications were similar between the two cohorts (*P* = 0.852; *P* = 0.527, respectively).

**Conclusions:**

Large randomized controlled trials are needed to robustly examine the comparative efficacy of these surgical options for lumbar spondylolisthesis. UBE-LIF appears advantageous in reducing operative time and improving surgical field exposure, which may potentially lower anesthesia-related risks and decrease anesthesia complications. Future large randomized controlled trials are needed to robustly examine the comparative efficacy of these techniques

## 1 Background

LSP primarily affects middle-aged and elderly populations (1) and is characterized by slippage of one vertebral body over another, which compromises spinal stability. This mechanical instability may impinge upon spinal nerves and vascular structures, producing symptoms such as lower back and radicular leg pain. In more advanced cases, neurological dysfunctions, including disturbances in bowel and bladder control, physical disability, and related sequelae, may occur (2, 3). Although conservative treatment may alleviate some symptoms, patients who respond poorly still require surgical intervention to restore spinal stability (4). Traditional posterior open lumbar decompression and fusion surgery is a classic treatment for this condition. However, its considerable invasiveness and associated damage to bones and ligaments result in slower postoperative recovery and potentially increase the risk of lumbar instability (5). A comparative orthopedic study by Moldovan et al. (6) reported that open reduction and internal fixation (ORIF) procedures were associated with significantly elevated levels of postoperative inflammatory markers compared to closed reduction internal fixation (CRIF), highlighting the importance of surgical invasiveness in postoperative systemic response. To reduce surgical trauma and promote patient recovery, minimally invasive techniques have gradually gained attention among spinal surgeons. Minimally invasive transforaminal lumbar interbody fusion (MIS-TLIF) has consistently demonstrated favorable clinical outcomes. Due to its reduced invasiveness and lower blood loss, MIS-TLIF has increasingly replaced traditional open surgery (7). Nevertheless, this technique requires retraction of surrounding soft tissues, potentially causing soft tissue damage. Additionally, the relatively narrow, fixed passage limits the surgical field of view (8). With the continuous advancement and widespread acceptance of endoscopic techniques and minimally invasive concepts, endoscopic fusion surgery is increasingly favored by orthopedic surgeons. Since Yeung (9) and Hoogland (10) introduced endoscopic lumbar discectomy and decompression procedures, percutaneous endoscopic (PE) techniques have rapidly advanced. These approaches further reduce damage to the posterior spinal structures and are highly valued by clinical practitioners. However, this method uses only a single composite metal cannula, limiting the surgical field and making it challenging to place larger spinal fusion devices. To overcome these limitations, De Antoni et al. (11) introduced the UBE approach. Compared to percutaneous endoscopic surgery, UBE provides independent operating portals, facilitating effective use of conventional surgical instruments. This method partially achieves the benefits of “minimally invasive open surgery,” significantly enhancing procedural flexibility and efficiency.

**Figure 1:**
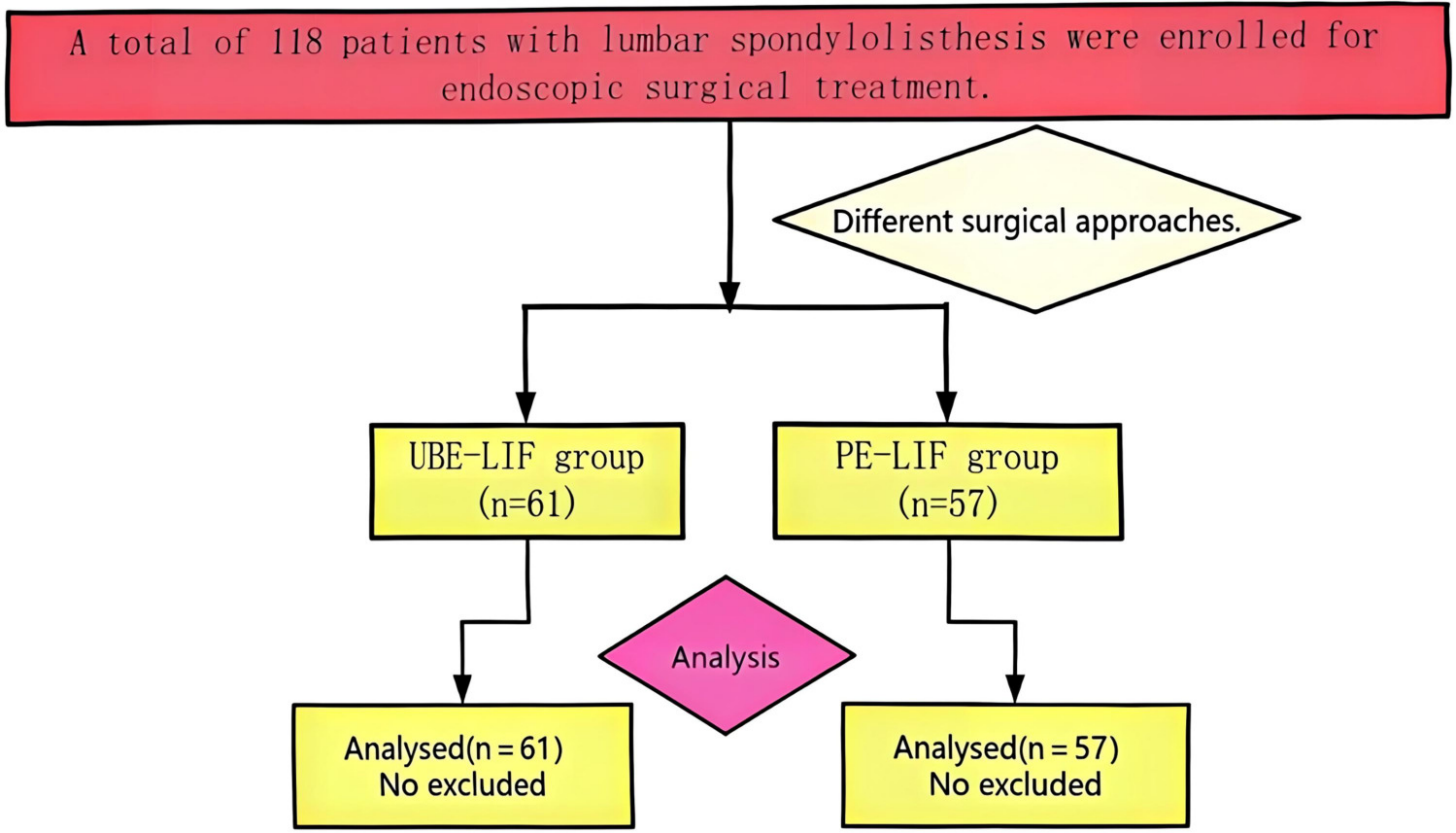
Patient screening flowchart.

However, few comparative studies exist between these two endoscopic techniques, either domestically or internationally (12, 13). Existing research evaluating these methods remains limited (14, 15). Continuous acquisition of clinical data remains essential for delineating the comparative strengths and weaknesses of these surgical techniques. In the present study, retrospective analyses were conducted on follow-up records of patients diagnosed with LSP who underwent either UBE-LIF or PE-LIF at our institution from June 2021 to August 2023. The objective was to evaluate and contrast the clinical effectiveness of these two methods.

## 2 Materials and methods

### 2.1 Inclusion and exclusion

Inclusion criteria: 1. Diagnosis of degenerative or isthmic LSP, with or without lumbar stenosis or disc herniation. 2. Single-segment LSP graded Meyerding I or II confirmed by imaging. 3. Ineffective conservative treatment lasting more than three months. 4. Postoperative follow-up of at least one year with complete imaging data.

Exclusion criteria: 1. Lumbar infections, tuberculosis, tumors, or other spinal disorders. 2. Congenital spinal deformities. 3. History of previous lumbar surgery. 4. Severe underlying medical or mental conditions that preclude surgery.

### 2.2 General information of the patients

Between June 2021 and August 2023, a total of 118 patients with single-segment LSP received surgical intervention through either UBE-LIF or PE-LIF at the Department of Minimally Invasive Spine Surgery, Sixth Affiliated Hospital of Xinjiang Medical University. Patients were categorized into two groups based on their respective surgical treatments: the UBE-LIF group (*n* = 61) and the PE-LIF group (*n* = 57). Each participant was monitored and evaluated for a duration of one year postoperatively (Figure 1). Ethical approval for this investigation was granted by the Ethics Committee of the Sixth Affiliated Hospital of Xinjiang Medical University (NO. LFYLLSC20241227-01).

### 2.3 Surgical method

#### 2.3.1 UBE-LIF

Under general anesthesia, each patient was placed in a prone position on the surgical table. For patients exhibiting asymmetrical clinical symptoms, fluoroscopic guidance via a C-arm was utilized to determine the intervertebral space on the symptomatic side. The entry point for puncture was marked roughly 0.5 cm lateral to the spinous process. After standard skin disinfection and sterile draping, a puncture needle established the UBE system under fluoroscopic guidance. The system was connected to a light source and camera, with color balance adjusted for optimal visualization. The endoscope was introduced through the working cannula, and fluid pressure was maintained at 30 mmHg by a pump.

A partial resection was performed on the medial side of the inferior articular facet of the superior vertebra and the medial side of the superior articular facet of the inferior vertebra, selectively removing part of the superior articular process. Following unilateral facetectomy, the nerve root was carefully mobilized medially with a UBE retractor introduced via the working portal. Herniated nucleus pulposus was subsequently extracted through the working portal using specialized forceps guided along the retractor. Neurodissectors were employed to dissect adhesions, and radiofrequency coagulation controlled bleeding. The nerve root was carefully freed and assessed for mobility, ensuring no nerve root or dural compression remained.

The intervertebral space was prepared, and autologous particulate bone grafts were packed alongside an appropriately sized carbon fiber cage, carefully adjusting position and orientation. Radiofrequency bipolar electrodes were utilized through the working channel to achieve hemostasis and ablation. Under fluoroscopic control, bilateral insertion of four pedicle screws was carried out. After removal of the working portal, thorough saline irrigation of the surgical site was performed, active bleeding points were meticulously verified, and a drainage tube was placed. Upon confirmation of accurate counts of surgical instruments and sponges, the wound was sutured layer by layer.

#### 2.3.2 PE-LIF

Following induction of general anesthesia, patients were positioned prone, and the surgical segment was confirmed and marked using a C-arm fluoroscopy system. A skin incision approximately 0.8 cm in length was made 2 cm lateral to the marked level to facilitate insertion of the working channel. Correct channel placement was verified under fluoroscopy, followed by connection of the spinal endoscopic system. With activation of the endoscope's light source, the device was introduced through the working channel, clearly visualizing the affected vertebral segment.

A radiofrequency knife was employed to carefully incise the ligamentum flavum, cautiously avoiding injury to the dural sac and nerve root. The working channel was then firmly secured within the intervertebral space. A ringsaw removed portions of the inferior articular process, and laminectomy procedures were conducted to expand the spinal canal. Bone-biting forceps, under endoscopic guidance, were utilized to remove hypertrophic bone tissue and residual ligamentum flavum posterior to the nerve root canal, ensuring decompression. Subsequently, the annulus fibrosus was incised, and nucleus pulposus was thoroughly removed with specialized forceps. The endplate was meticulously prepared using a semicircular sleeve and cutting saw to manage the cartilaginous endplate.

The harvested bone fragments, combined with 4 g of bone graft material, were compacted into an appropriately sized fusion device to preliminarily expand the intervertebral space. Under endoscopic visualization, the nerve root was assessed for damage or compression, and the correct positioning of the fusion device was confirmed. Hemostasis was achieved before withdrawing the working channel.

Pedicle punctures were performed bilaterally on the superior and inferior vertebrae utilizing puncture needles. After fluoroscopic confirmation of accurate placement, four separate incisions, approximately 1 cm each, were created at the puncture sites. Guidewires and cannulas were then positioned, facilitating bilateral insertion of four appropriately sized hollow pedicle screws into the upper and lower vertebral bodies. Correct screw placement was reconfirmed by fluoroscopy, followed by placement and tightening of connecting rods. Fluoroscopy subsequently confirmed successful reduction of the vertebral displacement and accurate positioning of screws and fusion device. The incision was repeatedly rinsed with saline, and active bleeding was assessed. After confirming that all surgical instruments and sponges were accounted for, the wound was closed in layers.

[All surgical procedures in both groups were performed by Director Xiangyu Meng (annual caseload >1,000 endoscopic spinal surgeries) to reduce potential bias from operator variability and inexperience.]

### 2.4 Postoperative management

Postoperative management was identical for both patient groups. Routine prophylactic antibiotics were administered preoperatively and continued through the first postoperative day. A pain pump was utilized for 48 h; following its removal, patients transitioned to oral acetaminophen with dihydrocodeine for pain relief. Patients were assisted to wear a lumbar brace and mobilize on the second postoperative day. Before discharge, lumbar spine anteroposterior and lateral x-rays, as well as lumbar CT with three-dimensional reconstruction, were performed. At each follow-up visit post-discharge, lumbar x-rays were re-evaluated, and lumbar CT with three-dimensional reconstruction was performed as needed. Patients were instructed to wear a lumbar brace for three months.

### 2.5 Clinical evaluation indicators

Surgical duration, estimated intraoperative blood loss, postoperative serum markers on day 3, surgical complications, and lumbar spine imaging results at the final follow-up were recorded and compared. Intervertebral fusion was assessed according to the Bridwell fusion grading criteria (16), with Grades I–II representing definitive fusion and Grades III–IV indicating possible or poor fusion. The VAS for lumbar and leg pain and the ODI scores were recorded at baseline and at 3 days, 3 months, 6 months, and 1 year postoperatively.

#### 2.5.1 VAS score

The VAS quantifies pain severity on a scale ranging from 0 (indicating the absence of pain) to 10 (the most severe pain imaginable). Patients rate their discomfort according to the following scale: 0 indicates absence of pain; scores from 1 to 3 denote mild pain that patients can tolerate; scores from 4 to 6 reflect moderate pain, sufficient to disrupt sleep yet still bearable; and scores from 7 to 10 represent severe pain, which is intolerable and significantly disrupts sleep and appetite.

#### 2.5.2 ODI score

The ODI questionnaire contains 10 questions, each covering areas. Each question has 6 response options scored from 0 to 5 points. The total score is calculated as follows:

ODIscore=(Totalobtainedpoints/(5×numberofquestionsanswered))×100,

with higher scores indicating greater functional impairment (maximum score: 100).

#### 2.5.3 Intraoperative blood loss calculation

Estimated blood loss = intraoperative suction volume − irrigation fluid volume.

#### 2.5.4 Fusion rate calculation

Fusion rate = (Grade I + Grade II cases)/(total number of cases).

### 2.6 Statistical methods

Statistical analysis was performed using SPSS 27.0 (International Business Machines Corporation, IBM; Armonk, New York, United States). Continuous variables are expressed as mean ± standard deviation $\left(\bar{x}\pm s\right)$; normality was assessed via Kolmogorov–Smirnov tests and normal distribution plots, with normally distributed data analyzed by independent samples *t*-tests and non-normal data by Mann–Whitney tests. Inter-group differences were compared using independent *t*-tests; intra-group temporal data employed paired *t*-tests or ANOVA, substituted by Wilcoxon signed-rank tests for non-normal distributions. Categorical data used chi-square or Fisher's exact tests; ordinal data utilized Mann–Whitney *U* tests; repeated measures employed Friedman tests, with statistical significance defined at *P* < 0.05.

## 3 Results

### 3.1 Participant analysis

A total of 118 patients diagnosed with LSP undergoing endoscopic surgery were included. Patients were divided into UBE-LIF (*n* = 61) and PE-LIF (*n* = 57) groups based on the surgical technique. All participants completed surgery and follow-up without loss. Postoperative complications included lower limb numbness (2 cases in PE-LIF; 1 case in UBE-LIF) and cerebrospinal fluid (CSF) leakage (1 case in PE-LIF; 3 cases in UBE-LIF). Patients experiencing lower limb numbness received dexamethasone sodium phosphate and mannitol injection for symptom relief. Patients with CSF leaks were placed in a head-down position until drainage decreased below 30 ml/day. Ultimately, symptoms resolved in all 7 patients, enabling discharge. No patients experienced cauda equina injury, fusion device subsidence or displacement, or severe complications such as hematoma or incision infection.

### 3.2 Comparison of preoperative data between the two groups

No significant differences were observed between groups in age, surgical segments, gender distribution, or hospitalization duration (*P* > 0.05). Preoperative VAS scores, ODI scores, and serum markers also showed no significant differences (*P* > 0.05) (Table 1).

**Table 1:** Baseline data of the two groups of patients before surgery.

Variables	Total (*n* = 118)	PE-LIF (*n* = 57)	UBE-LIF (*n* = 61)	Statistic	*P*
Age Y, $\left(\bar{x}\pm s\right)$	61.11 ± 10.95	60.96 ± 11.63	61.26 ± 10.26	*t* = −0.146	0.884
Sex, *n* (%)				*χ*2 = 0.703	0.402
Female	72 (61.017)	37 (64.91)	35 (57.37)		
Men	46 (38.983)	20 (35.08)	26 (42.62)		
Operative segment, *n* (%)				*χ*^2^ =0.206	0.902
L5/S1	19 (16.102)	9 (15.78)	10 (16.39)		
L3/L4	9 (7.627)	5 (8.77)	4 (6.55)		
L4/L5	90 (76.271)	43 (75.43)	47 (77.04)		
Hospitalization time (d), $\left(\bar{x}\pm s\right)$	13.78 ± 2.90	14.24 ± 2.96	13.34 ± 2.76	*t* = 1.692	0.093
Pre-op VAS back pain score, $\left(\bar{x}\pm s\right)$	4.82 ± 1.15	4.87 ± 1.33	4.77 ± 0.94	*t* = 0.492	0.624
Pre-op VAS leg pain score, $\left(\bar{x}\pm s\right)$	6.27 ± 1.14	6.35 ± 1.22	6.19 ± 1.06	*t* = 0.725	0.470
Pre-op ODI, $\left(\bar{x}\pm s\right)$	64.25 ± 8.00	64.49 ± 8.62	64.03 ± 7.36	*t* = 0.308	0.758
Pre-op CRP, $\left(\bar{x}\pm s\right)$	1.86 ± 2.35	2.02 ± 1.94	1.71 ± 2.66	*t* = 0.711	0.478
Pre-op CK, $\left(\bar{x}\pm s\right)$	75.13 ± 29.50	77.89 ± 33.80	72.55 ± 24.53	*t* = 0.979	0.330

### 3.3 Comparison of postoperative VAS scores and ODI scores between the two groups

**Figure 2:**
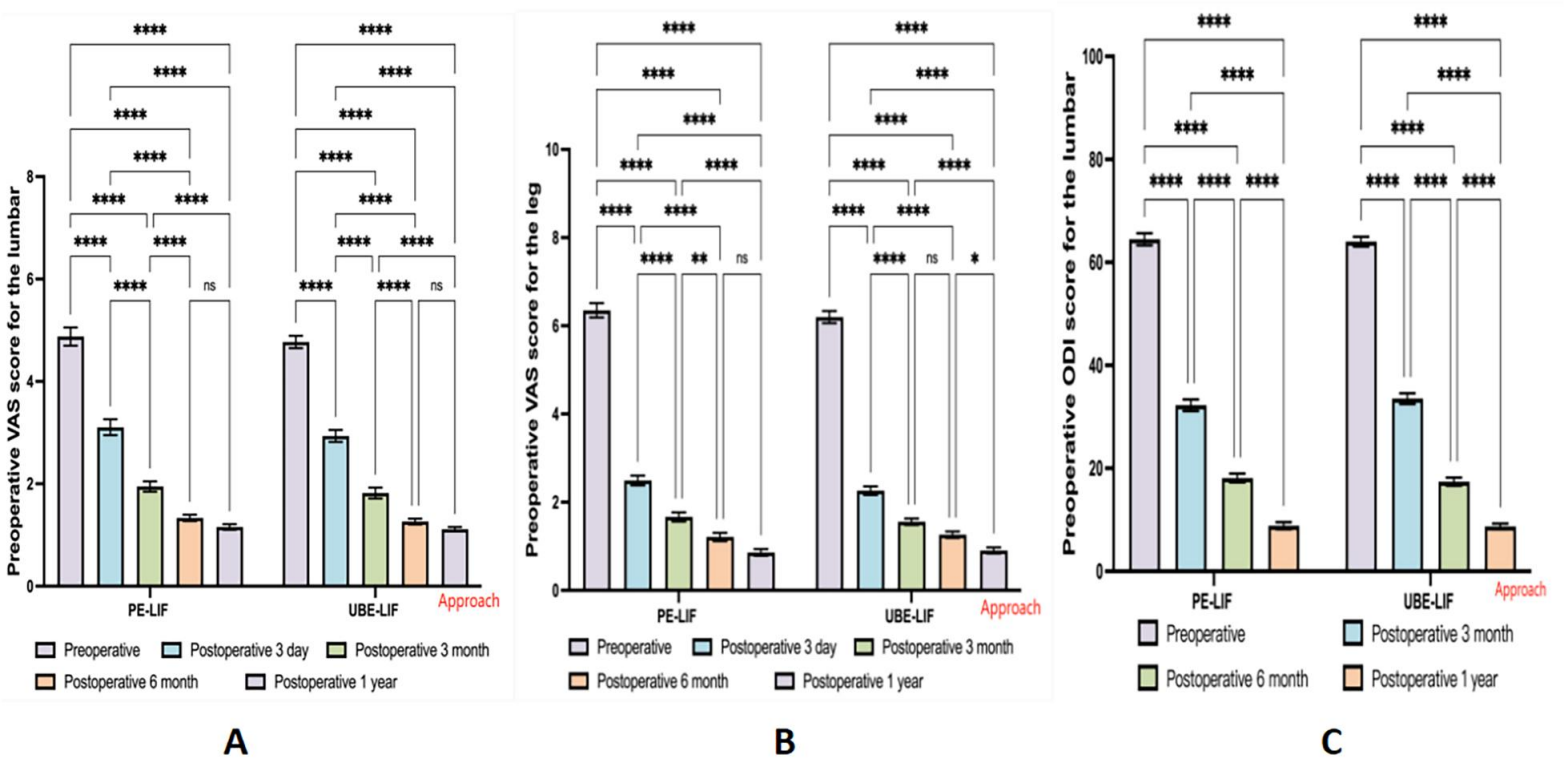
Bar chart of changes in VAS and ODI before and after surgery. **(A)** Changes in back VAS scores for two surgical procedures at different time points. **(B)** Changes in leg VAS scores for two surgical procedures at different time points. **(C)** Changes in ODI scores for two surgical procedures at different time points. **p < 0.05; ****p < 0.01; ns *p* > 0.05.

Compared to their preoperative assessments, both patient groups showed statistically significant improvement in VAS scores for back pain, leg pain, and ODI scores across all postoperative follow-up periods. Notably, VAS scores demonstrated pronounced improvement within the early postoperative phase (3 days to 3 months). However, comparisons between groups revealed no statistically significant differences at any postoperative assessment point (all *P* > 0.05). These outcomes are detailed in Table 2, with Figures illustrating changes in back pain (Figure 2A), leg pain (Figure 2B), and ODI scores (Figure 2C).

**Table 2:** Postoperative VAS and ODI score indicators.

Variables	Follow-up	PE-LIF (*n* = 57)	UBE-LIF (*n* = 61)	Statistic	*P*
Back pain VAS $\left(\bar{x}\pm s,\mathrm{scores}\right)$	After-op 3d	3.10 ± 1.16	2.93 ± 0.92	*t* = 0.879	0.381
	After-op 3m	1.94 ± 0.75	1.82 ± 0.82	*t* = 0.869	0.387
	After-op 6m	1.33 ± 0.47	1.26 ± 0.44	*t* = 0.840	0.403
	After-op 1y	1.15 ± 0.41	1.11 ± 0.31	*t* = 0.635	0.527
Leg pain VAS $\left(\bar{x}\pm s,\mathrm{scores}\right)$	After-op 3d	2.49 ± 0.81	2.26 ± 0.74	*t* = 1.577	0.118
	After-op 3m	1.66 ± 0.78	1.55 ± 0.55	*t* = 0.872	0.385
	After-op 6m	1.21 ± 0.69	1.26 ± 0.57	*t* = −0.440	0.661
	After-op 1y	0.86 ± 0.57	0.90 ± 0.53	*t* = −0.408	0.684
ODI $\left(\bar{x}\pm s,\mathrm{scores}\right)$	After-op 3m	32.24 ± 8.58	33.54 ± 8.04	*t* = −0.839	0.403
	After-op 6m	18.12 ± 6.39	17.41 ± 6.21	*t* = 0.609	0.544
	After-op 1y	8.86 ± 5.04	8.70 ± 4.35	*t* = 0.177	0.860

### 3.4 Comparison of surgical and serological indicators between the two groups

The operation time was significantly prolonged in the PE-LIF group compared with the UBE-LIF group (*P* < 0.001, Table 3). Although intraoperative blood loss was somewhat higher in the PE-LIF cohort, this difference did not reach statistical significance (*P* = 0.258, Table 3). On postoperative day 3, patients who underwent UBE-LIF demonstrated significantly lower CRP levels than those treated with PE-LIF (*P* = 0.009, Table 3). However, CK levels showed no significant difference between the two groups (*P* = 0.258, Table 3).

**Table 3:** Surgical-related and laboratory test indicators.

Variables	PE-LIF (*n* = 57)	UBE-LIF (*n* = 61)	Statistic	*P*
Surgical time $\left(\min,\bar{x}\pm s\right)$	227.05 ± 56.08	179.75 ± 42.96	*t* = 5.073	<0.001
Intraoperative bleeding volume $\left(\mathrm{ml},\bar{x}\pm s\right)$	177.89 ± 55.89	170.90 ± 74.76	*t* = 0.572	0.568
After-op3d CRP, $\left(\bar{x}\pm s\right)$	82.09 ± 51.84	57.88 ± 46.49	*t* = 2.652	0.009
After-op3d CK, $\left(\bar{x}\pm s\right)$	423.65 ± 97.73	443.64 ± 91.54	*t* = −1.137	0.258

### 3.5 Comparison of complications and fusion rates between the two groups

even complications were documented across both groups. Specifically, in the PE-LIF group, there were two incidents of lower limb numbness and one CSF leakage. Conversely, the UBE-LIF group reported one instance of lower limb numbness and three incidents of CSF leakage. Statistical analysis indicated no significant difference in complication rates between the two cohorts (*P* = 0.527, Table 4).

**Table 4:** Complications and fusion rate.

Variables	Total (*n* = 118)	PE-LIF (*n* = 57)	UBE-LIF (*n* = 61)	Statistic	*P*
Complication, *n* (%)				*χ*^2^ =1.28	0.527
Nothing	111 (94.1)	54 (94.7)	57 (93.4)		
Exist	7 (5.9)	3 (5.3)	4 (6.5)		
Fusing, *n* (%)				*χ*^2^ =0.384	0.852
I grade	95 (80.5)	46 (80.7)	49 (80.3)		
II grade	12 (10.2)	5 (8.8)	7 (11.5)		
III grade	11 (9.3)	6 (10.5)	5 (8.2)		
IV grade	0 (0.0)	0 (0.0)	0 (0.0)		

At the 1-year follow-up, fusion rates between groups did not differ significantly (*P* = 0.852, Table 4). Fusion success rates (Grades I and II) were recorded as 91.8% for patients in the UBE-LIF group and 89.5% in the PE-LIF group.

### 3.6 Typical cases

#### 3.6.1 PE-LIF

**Figure 3:**
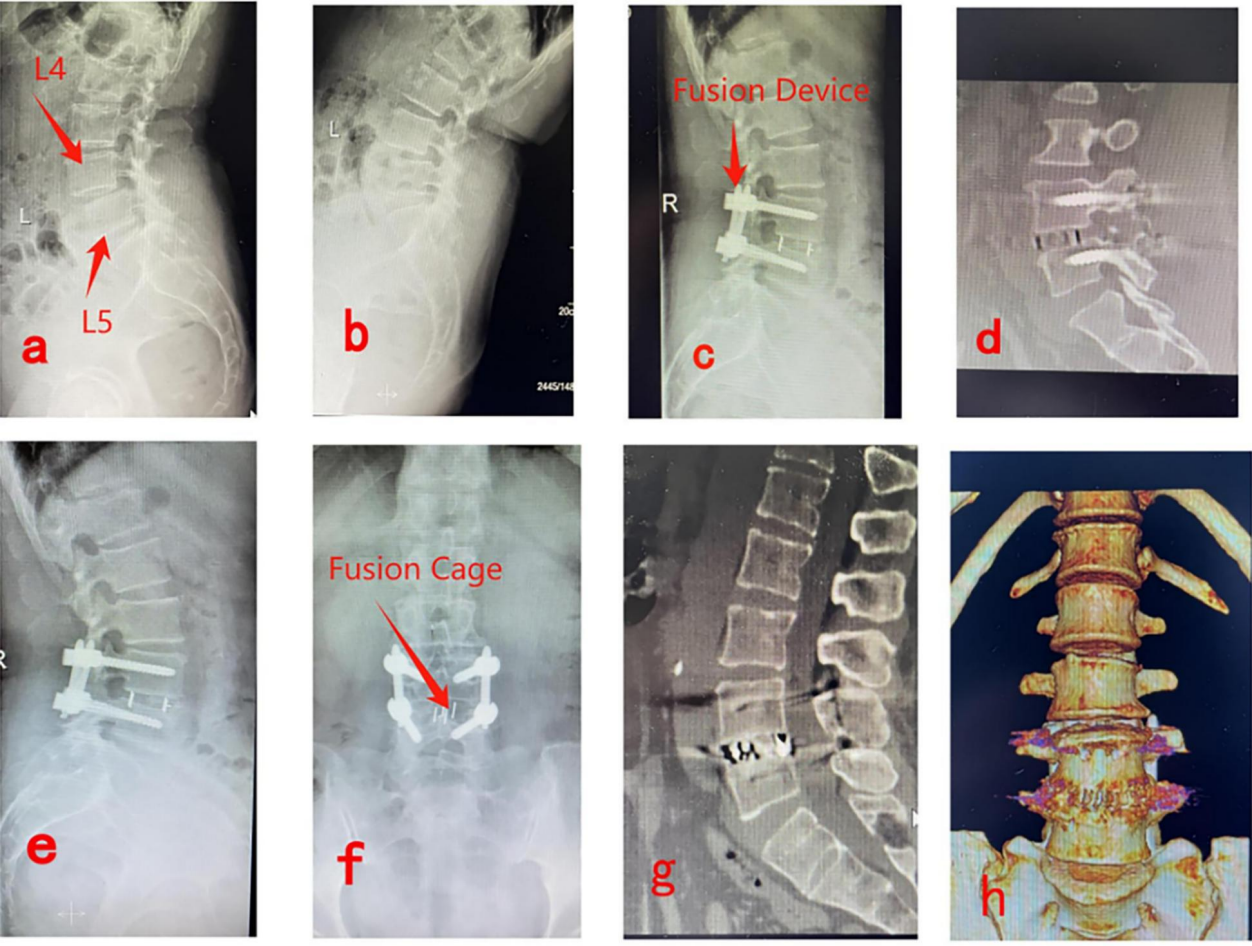
Pre- and post-operative imaging data for PE-LIF. **(a,b)** Show preoperative lateral x-rays indicating L4/5 LSP; **(c,d)** illustrate x-ray and CT images 3 days postoperatively, showing adequate interbody bone grafting and proper positioning of the fusion device; **(e,f)** demonstrate lateral x-rays 6 months postoperatively, indicating satisfactory vertebral body reduction, no internal fixation breakage, and no fusion device subsidence; **(g,h)** show CT and three-dimensional reconstruction images 1 year postoperatively, confirming satisfactory vertebral reduction and bony fusion between vertebral bodies.

Figures 3a,b show preoperative lateral x-rays indicating L4/5 LSP; Figures 3c,d illustrate x-ray and CT images 3 days postoperatively, showing adequate interbody bone grafting and proper positioning of the fusion device; Figures 3e,f demonstrate lateral x-rays 6 months postoperatively, indicating satisfactory vertebral body reduction, no internal fixation breakage, and no fusion device subsidence; Figures 3g,h show CT and three-dimensional reconstruction images 1 year postoperatively, confirming satisfactory vertebral reduction and bony fusion between vertebral bodies (Figure 3).

#### 3.6.2 UBE-LIF

**Figure 4:**
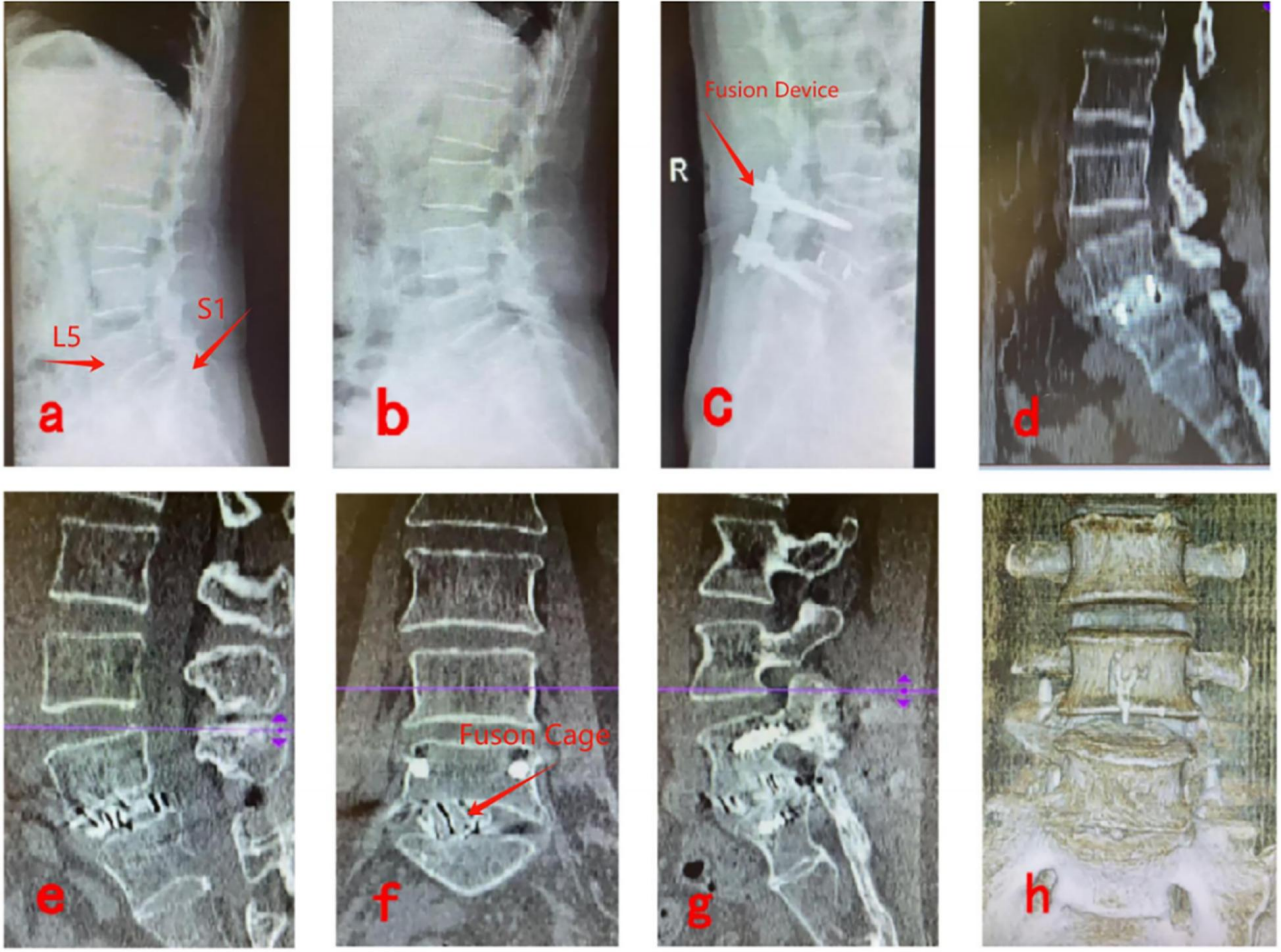
Pre- and post-operative imaging data for UBE-LIF. **(a,b)** Show preoperative lateral x-rays indicating L5/S1 LSP; **(c,d)** display x-ray and CT images 3 days postoperatively, showing proper positioning of the fusion device and no breakage of rods or screws; **(e,f)** present CT images 6 months postoperatively, confirming effective vertebral body reduction and no fusion device subsidence; **(g,h)** illustrate CT and three-dimensional reconstruction images 1 year postoperatively, verifying satisfactory vertebral reduction and definitive bony fusion between vertebral bodies.

Figures 4a,b show preoperative lateral x-rays indicating L5/S1 LSP; Figures 4c,d display x-ray and CT images 3 days postoperatively, showing proper positioning of the fusion device and no breakage of rods or screws; Figures 4e,f present CT images 6 months postoperatively, confirming effective vertebral body reduction and no fusion device subsidence; Figures 4g,h illustrate CT and three-dimensional reconstruction images 1 year postoperatively, verifying satisfactory vertebral reduction and definitive bony fusion between vertebral bodies (Figure 4).

## 4 Discussion

LSP is a common spinal disorder causing lower back and leg pain and numbness. For patients who respond poorly to conservative treatment, interbody fusion surgery represents an effective therapeutic approach. Although traditional posterior open fusion surgery provides satisfactory clinical outcomes, significant damage to paravertebral soft tissues and bony structures frequently occurs, increasing risks of postoperative complications and infections. With advancements in minimally invasive techniques, endoscopic lumbar interbody fusion surgery has gained increasing acceptance among spinal surgeons. Compared to traditional open surgery, endoscopic procedures better preserve vertebral structures, thus reducing surgical trauma, shortening operation duration, and facilitating postoperative recovery (17, 18).

However, percutaneous endoscopic surgery uses a single metal tube, resulting in certain limitations. These limitations include restricted surgical flexibility, a relatively narrow surgical field of view, and inadequate control over bleeding and decompression. Additionally, the procedure places higher demands on surgical instruments, complicating adequate decompression and fusion (19). In the 1990s, De Antoni et al. (11) initially introduced a surgical approach using the UBE technique. Subsequently, in 2016, Heo et al. (20) successfully performed the first lumbar interbody fusion using the UBE approach, achieving favorable clinical outcomes. Compared with percutaneous endoscopy, UBE provides greater surgical flexibility and efficiency due to independent operating channels. Furthermore, UBE combines the visualization advantages of open surgery with the minimal invasiveness of endoscopic procedures, effectively achieving minimally invasive open surgery.

Specifically, the UBE technique utilizes two distinct surgical access points positioned ipsilaterally: one dedicated to visualization and the other for operative procedures. These dual channels function independently, avoiding mutual interference (21). Recent advancements in endoscopic methods have contributed to UBE-LIF emerging as a feasible alternative to conventional fusion operations. This method overcomes certain drawbacks inherent in percutaneous endoscopic techniques and is being increasingly adopted in treating various spinal conditions, encompassing cervical and thoracic pathologies.

Our findings demonstrated considerable postoperative enhancements in VAS scores for both low back and leg pain, as well as ODI scores at all follow-up intervals relative to preoperative values (Table 2). Figures 2A–C illustrate that the VAS and ODI scores showed substantial reductions during the initial 3 months after surgery. Subsequent improvements, although persistent, proceeded at a slower pace after the initial postoperative phase. This implies that both surgical approaches effectively alleviate acute postoperative pain and support early functional rehabilitation. Nevertheless, intergroup comparisons revealed no statistically significant differences at any follow-up point after surgery (*P* > 0.05, Table 2), underscoring the equivalent clinical outcomes of UBE-LIF and PE-LIF regarding pain alleviation and functional restoration. Furthermore, duration of hospital stay was similar between the two groups (*P* = 0.093, Table 1), highlighting comparable efficacy and safety profiles.

Surgical duration differed significantly between the two techniques, favoring UBE-LIF (*P* < 0.001, Table 3). However, no significant difference in estimated intraoperative blood loss was found. Surgeons' proficiency and preferences regarding each method may influence these metrics. Additionally, the dual-port design of UBE theoretically involves more muscle dissection compared with the single-port approach, potentially increasing blood loss.

In reviewing relevant literature, a retrospective analysis by Fan et al. (22) reported higher intraoperative blood loss in the UBE group compared to the PE group (*P* < 0.001). However, a subsequent study by the same researchers found less blood loss with UBE compared to PE (23). Two additional studies (24, 25) indicated lower average blood loss with UBE-LIF than PE-LIF, although none reported statistically significant differences, aligning with the present study.

Possible explanations for these findings include: 1. UBE surgery provides greater flexibility with clearer and broader visualization during the procedure; 2. a wider selection of instruments in UBE facilitates more effective hemostasis and decompression, reducing blood loss and operative time; 3. the longer duration of PE-LIF may increase blood loss; and 4. different methods used to calculate blood loss across studies might lead to varying results. Future research should quantify hidden blood loss and hemoglobin reduction to more precisely evaluate intraoperative blood loss between techniques.

Serum CK levels can reflect muscle injury extent following spinal surgery (26). Studies have shown a close correlation between CK levels and paravertebral muscle damage (27). CRP is a sensitive marker of inflammation, infection, and tissue injury, typically peaking around 3 days postoperatively (28). In the present study, both groups showed significant increases in CRP and CK levels at 3 days postoperatively. However, CRP was significantly lower in the UBE-LIF group than in the PE-LIF group (*P* = 0.009, Table 3). In contrast, no significant difference in CK levels was observed between groups (*P* = 0.258, Table 3).

Few comparative studies exist regarding postoperative CK and CRP levels between these two surgical techniques (22, 29), and existing reports show inconsistent findings. One study (30) comparing UBE-TLIF with MIS-TLIF found generally lower serum CK and CRP levels with UBE, suggesting less surgical trauma and muscle injury. However, the current study showed no significant difference in postoperative CK levels. Possible explanations include: 1. the broader operative field of UBE reduces operative time and blood loss, resulting in lower CRP changes at 72 h postoperatively, but the dual-portal approach necessitates larger incisions; 2. UBE employs larger instruments, potentially causing greater muscle trauma and accounting for the nonsignificant difference in CK levels; and 3. CK level fluctuations vary over time, and single sampling at 72 h postoperatively may introduce selection bias. Future studies will provide more detailed and rigorous analyses of muscle and soft-tissue injury associated with these surgical methods, enhancing clinical understanding of the UBE-LIF technique.

Additionally, the interbody fusion rate is an essential factor in lumbar fusion surgery. From a technical perspective, spinal endoscopy provides enhanced visualization, allowing accurate assessment of endplate preparation. This reduces the risk of subchondral bone damage to the endplates, preventing subsidence or fusion failure of the interbody device. In this study, both patient groups achieved satisfactory fusion outcomes, consistent with previous studies (23, 31). No significant difference in fusion rates was identified between the two surgical techniques at the 1-year follow-up (*P* = 0.852, Table 4), confirming comparable effectiveness. Nevertheless, a few patients in both groups experienced poor fusion outcomes at 1 year. Continuous patient monitoring and, if necessary, revision treatments are recommended.

The authors propose that, besides intervertebral infections, endplate preparation, and bone graft material characteristics, patient osteoporosis status may also impact fusion outcomes. Given the degenerative nature of LSP and the average patient age around 60 years, bone density evaluation before surgery is advisable. If indicated, osteoporosis treatment may further facilitate patient recovery.

Postoperative complications are another critical concern. All surgeries were completed successfully without severe perioperative complications such as cauda equina injury, fusion device subsidence or displacement, internal fixation breakage, nerve root injury, hematoma, or incision infection. A total of 7 early postoperative complications occurred, with no significant difference between groups (*P* > 0.05, Table 4). Patients experiencing lower limb numbness were treated with dexamethasone sodium phosphate and mannitol injections. Patients with CSF leaks remained in a head-down, foot-up position until drainage was below 30 ml/day. All 7 patients were discharged successfully without further complications.

Lower limb numbness and CSF leaks are common complications of endoscopic spinal surgery. These issues may result from surgeons' familiarity with surgical techniques and patients' degree of spinal degeneration. Early in the learning curve, unclear endoscopic visualization combined with severe degenerative changes can increase nerve or dural injuries. The authors recommend surgeons gain thorough anatomical understanding and proceed cautiously during surgery. Additionally, less-experienced surgeons should receive guidance from senior colleagues to reduce intraoperative complications effectively.

In summary, the authors suggest that UBE-LIF offers several advantages compared to PE-LIF: 1. Independent operating and viewing channels remove instrument-use limitations, increasing surgical flexibility, enabling precise decompression, and reducing operative time (32). 2. A broader surgical field allows for more comprehensive decompression and harvesting of greater amounts of autologous bone, thus increasing fusion success. 3. UBE-LIF is easy to master, with a shorter learning curve. 4. Increased operative flexibility and fewer instrument constraints improve bleeding control, thereby reducing intraoperative blood loss.

Compared to UBE, PE-LIF offers greater minimally invasive advantages, resulting in less muscle dissection and damage, facilitating faster incision healing. No significant differences were observed between the two methods regarding fusion rates and complications. Based on current data, the authors recommend UBE-LIF for older patients with multiple comorbidities and severe lumbar or leg symptoms when both methods are feasible, as shorter operative times reduce perioperative cardiovascular risks and economic burdens, while lower postoperative CRP levels suggest smoother inflammatory responses and faster recovery.

However, This lower-level clinical retrospective study lacks randomization to reduce bias; moreover, the advanced mean age of included patients with varying degrees of osteoporosis or cardiovascular comorbidities—without stratification—may introduce fusion assessment errors. Thirdly, intraoperative saline irrigation potentially confounds blood loss quantification. Fourthly, absent spondylolisthesis-grade subgrouping risks outcome bias. Fifthly, single-surgeon expertise (>1,000 cases/year) limits generalizability to less-experienced practitioners. Sixthly, 12-month follow-up precludes long-term fusion/complication evaluation.

Future research should expedite ethical approvals and include multicenter randomized controlled trials (RCTs), extended follow-up periods, standardized blood-loss assessment protocols, and surgeon-experience stratification to clearly define the UBE learning curve and provide robust clinical evidence.

## 5 Conclusion

Preliminary analysis indicates that both UBE-LIF and PE-LIF represent viable surgical techniques for lumbar spondylolisthesis. UBE-LIF demonstrates potential advantages in maneuverability, intraoperative field visualization, operative duration, and blood loss control, while the single-port design of PE-LIF may contribute to a potentially less invasive profile. It must be emphasized that these retrospective analyses cannot evaluate the non-inferiority of either technique. In clinical practice, surgeons may select an approach based on individual patient characteristics and technical expertise to optimize endoscopic surgery benefits—such as reducing patient discomfort and complication risks—though these conclusions remain preliminary findings requiring validation through larger-scale prospective studies.

## Data Availability

The original contributions presented in the study are included in the article/Supplementary Material, further inquiries can be directed to the corresponding author.
